# How Does Fertility Stress Influence Depressive Symptoms in Female Partners of Infertile Couples in China? A Parallel Mediation Analysis of Infertility Stigma and Family Function

**DOI:** 10.1155/da/5703300

**Published:** 2026-06-27

**Authors:** Yingqi Li, Ying Wang, Meihong Wang, Hua Yang, Lei Qiu

**Affiliations:** ^1^ Key Laboratory of Tropical Translational Medicine of Ministry of Education, School of Public Health, Hainan Academy of Medical Sciences, Hainan Medical University, Haikou, 571199, Hainan, China, hainmc.edu.cn; ^2^ Hainan Provincial Key Laboratory for Human Reproductive Medicine and Genetic Research, The First Affiliated Hospital of Hainan Medical University, Hainan Medical University, Haikou, 571101, Hainan, China, hainmc.edu.cn; ^3^ Department of Gynecology, Hainan Women and Children’s Medical Center, Haikou, 571101, Hainan, China

**Keywords:** depressive symptoms, family function, fertility stress, infertility, infertility stigma

## Abstract

**Objective:**

This study aims to explore the impact of fertility stress on depressive symptoms, with a focus on analyzing the potential mediating effects of infertility stigma and family function in this process.

**Methods:**

A cross‐sectional design was employed in which 1294 female partners of infertile couples were recruited from two tertiary‐grade A hospitals in Hainan Province, China. Fertility stress, depressive symptoms, infertility stigma, and family function were assessed with validated self‐report questionnaires. Structural equation modeling (SEM) was used to estimate the direct effect of fertility stress and the parallel mediating effects of infertility stigma and family function.

**Results:**

The prevalence rate of depressive symptoms was 26.35%. The direct effect model indicated a significant positive association between fertility stress and depressive symptoms (*β* = 0.260, *p* < 0.001). The parallel mediation model revealed that infertility stigma (*β* = 0.176, *p* < 0.001) and family function (*β* = 0.052, *p* < 0.001) exerted full mediating effects. Specifically, fertility stress indirectly increased depressive symptoms by exacerbating infertility stigma; concurrently, low levels of family function exacerbated the negative impact of fertility stress on mental health.

**Conclusion:**

Fertility stress not only directly affects depressive symptoms, but also indirectly exacerbates depressive symptoms by intensifying infertility stigma and impairing family function. This study provides a new perspective for understanding the mental health mechanism in female partners of infertile couples, emphasizing that psychological interventions need to simultaneously focus on reducing infertility stigma and improving family function, which may offer a basis for informing relevant clinical practices and public health policies.

## 1. Introduction

Infertility, defined as the inability of a couple to achieve pregnancy after 1 year of regular unprotected intercourse, has become a major global public health issue [1], with profound implications for women’s mental health. According to the World Health Organization, ~17.5% of the global adult population is affected by infertility, and this proportion has been increasing annually [2]. Similar to global trends, China is facing a rising prevalence of infertility, which has increased from 12% to 18% in recent years [3].

Infertility not only impairs reproductive capacity but also exerts significant negative impacts on women’s mental health, particularly in socio‐cultural contexts that strongly emphasize fertility. In traditional Chinese culture, fertility is regarded as an essential familial responsibility for the continuation of the family lineage [4]. Consequently, women experiencing infertility face not only internalized self‐perception pressure but also intense societal judgments and family expectations. These cultural and social pressures may contribute to elevated fertility stress, which has been shown to have substantial and lasting implications for women’s mental health [5, 6].

Infertility also affects the couple as a relational unit and may reshape the broader family environment. As a shared and often chronic stressor, infertility can disrupt marital communication, increase emotional tension between partners, and weaken mutual support, particularly when repeated treatment failures or prolonged uncertainty are involved [7]. In family‐centered cultural settings such as China, infertility may also trigger pressure from parents and extended family members regarding childbearing, thereby further straining family relationships and daily functioning [8]. These interpersonal and family‐contextual consequences suggest that the psychological impact of infertility should be understood not only at the individual level but also within the couple and family system.

A growing body of research has reported a positive association between fertility stress and depressive symptoms, particularly among infertile women in China [9]. Infertile women have been found to be at elevated risk for depressive symptoms, with reported prevalence estimates exceeding 21% [10]. However, much of the existing literature has focused on direct associations using conventional regression approaches, with less attention to the psychosocial mechanisms through which fertility stress may be linked to depressive symptoms. A clearer understanding of these mechanisms is important for identifying potential targets for psychological support and intervention.

The stress‐coupling Model proposed by Lazarus and Folkman provides a useful framework for understanding the dynamic interplay among these variables [10, 11]. Within this framework, fertility stress can be understood as a salient stressor for infertile women, shaped by personal expectations, sociocultural norms, family dynamics, and treatment‐related burdens [12]. When coping resources are insufficient, persistent fertility stress may be associated with greater vulnerability to depressive symptoms [13]. Guided by this framework, the present study examines whether infertility stigma and family function may help explain the association between fertility stress and depressive symptoms, with fertility stress conceptualized as the primary stressor and infertility stigma as a psychosocial response within the infertility context.

Infertility stigma is another important psychosocial factor associated with the mental health of infertile women. Rooted in the work of Goffman [14], stigma refers to the social devaluation and exclusion experienced by individuals with a stigmatized condition, which can elicit feelings of shame, inferiority, and social withdrawal, thereby increasing the risk of depressive symptoms [14, 15]. Infertile women often face negative labeling and social judgment because of their inability to conceive, which may intensify feelings of shame, self‐blame, and social withdrawal [16]. This stigma may arise not only after a formal diagnosis of infertility but also from broader social expectations regarding marriage, motherhood, and childbearing. In the Chinese cultural context, women may continue to bear disproportionate social pressure even when infertility is primarily attributable to male factors [17]. Studies by Taebi et al. [18] and Zhao et al. [19] have demonstrated that higher levels of infertility stigma are associated with more negative self‐perceptions, greater self‐blame, increased social withdrawal, and heightened risk of depressive symptoms among infertile women. This variable may function as a mediator in the relationship between fertility stress and depressive symptoms.

Family function is also likely to be relevant in the context of infertility as infertility often unfolds within close couple interactions and broader family relationships. Effective family function may provide emotional support, practical assistance, and adaptive communication, thereby helping women cope with infertility‐related stress [20, 21]. Conversely, infertility may strain family relationships, disrupt communication, and reduce perceived support, which may, in turn, be associated with poorer psychological adjustment [22, 23]. These findings suggest that family function may also serve as a mediator in the association between fertility stress and depressive symptoms.

Existing studies suggest that fertility stress may be associated with depressive symptoms not only directly but also indirectly through interpersonal and psychosocial pathways. In particular, infertility stigma and family function have each been shown to be related to the mental health of infertile women [24–28]. However, these two factors have rarely been examined simultaneously within a single analytical framework. As a result, the extent to which infertility stigma and family function operate as parallel mediators in the association between fertility stress and depressive symptoms remains unclear.

To address this gap, the present study employs a parallel mediation model to examine whether infertility stigma and family function mediate the association between fertility stress and depressive symptoms among female partners of infertile couples. This approach allows the simultaneous evaluation of multiple mediators and their relative contributions [29]. Clarifying these pathways may help inform more targeted psychosocial support for infertile women.

## 2. Conceptual Model and Hypotheses

**Figure 1:**
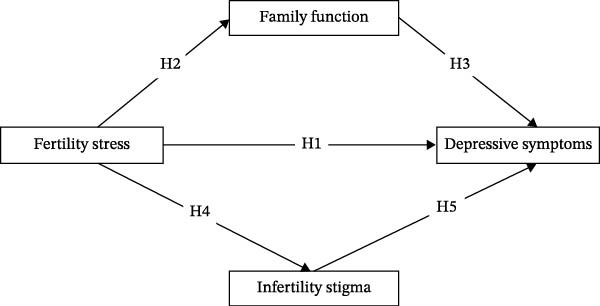
Conceptual model of the associations among fertility stress, infertility stigma, family function, and depressive symptoms.

Based on prior literature, fertility stress, infertility stigma, family function, and depressive symptoms appear to be closely interconnected among infertile women. Fertility stress has been associated with greater depressive symptoms, higher infertility stigma, and poorer family function [13, 19, 22]. In turn, both infertility stigma and family function have been linked to depressive symptoms [19, 22]. On this basis, we proposed a conceptual model in which infertility stigma and family function operate as parallel mediators of the association between fertility stress and depressive symptoms (Figure 1).

H1: Fertility stress is positively associated with depressive symptoms.

H2: Fertility stress is negatively associated with family function.

H3: Family function is negatively associated with depressive symptoms.

H4: Fertility stress is positively associated with infertility stigma.

H5: Infertility stigma is positively associated with depressive symptoms.

## 3. Methods

### 3.1. Study Sample

This cross‐sectional study was conducted at the reproductive medicine centers of two Grade A tertiary hospitals (The First Affiliated Hospital of Hainan Medical University and Hainan Women and Children’s Medical Center) in Haikou, Hainan Province, from July 2023 to February 2024. The study focused on female partners of infertile couples because the primary outcome was depressive symptoms in women and because women often bear substantial infertility‐related stigma, family pressure, and psychological burden in the Chinese cultural context. The inclusion criteria were: (1) age ≥18 years; (2) willingness to participate in the study after providing informed consent; and (3) ability to read and write to independently complete the questionnaires. The exclusion criteria were: (1) history of mental illness or cognitive impairment; (2) hearing or language barriers that prevented questionnaire completion; and (3) Withdrawal from the study during the research process.

### 3.2. Data Collection and Ethical Review

Eligible female participants were recruited during their clinical visits to the reproductive medicine centers. After enrollment, researchers administered questionnaires covering general demographic information, infertility‐related clinical characteristics, and survey scales. The questionnaires were completed through face‐to‐face interviews with the assistance of nurses from the reproductive medicine centers. Because the study involved sensitive infertility‐related topics, reproductive nurses were involved in recruitment and questionnaire administration. Before data collection, they received standardized training on study procedures, informed consent, questionnaire administration, communication regarding sensitive questions, and confidentiality requirements to ensure consistency in data collection. All questionnaires were checked immediately after completion to ensure accuracy and completeness, and all research data were kept strictly confidential. The study was approved by the Ethics Committees of the Infertility Centers of both hospitals (approval number 2023‐KYL‐075) and adhered to the ethical principles of the Declaration of Helsinki. Written informed consent was obtained from all participants prior to their involvement in the survey.

### 3.3. Sample Size and Response Rate

When a structural equation model contains multiple indicators per latent variable, the minimum sample size should be at least 10 times the number of observed items [30]. Given that the combined scales comprised 100 items, the required minimum was 100 × 10 = 1000 participants. A total of 1338 questionnaires were returned; after excluding invalid responses (e.g., random responding, incomplete data, or repetitive patterns), 1294 valid cases from female partners of infertile couples were retained, yielding an effective response rate of 96.71%. This final sample size met the recommended threshold.

### 3.4. Instruments

#### 3.4.1. General Information Questionnaire

A self‐constructed general questionnaire was used to collect basic sociodemographic data from female partners of infertile couples. Items included age, ethnicity, residence, native place, educational level, and self‐perceived economic pressure. We also assessed reproductive‐related variables, specifically the duration of infertility treatment and history of miscarriage.

#### 3.4.2. Fertility Problem Inventory (FPI)

To quantify fertility stress among female partners of infertile couples, researchers employed the FPI, originally developed by Newton et al. [27] Having been widely validated in the Chinese infertile population, its Chinese version demonstrates satisfactory reliability and validity. This 46‐item scale assesses five dimensions: social concern, marital relationship, sexual concern, need for parenthood, and rejection of childfree living [31]. Representative items address concerns about social pressure, strain in the marital relationship, discomfort related to sexual life, and the importance attached to becoming a parent. Responses to each item are rated on a 6‐point Likert scale (1 = strongly disagree and 6 = strongly agree), with higher scores indicating greater fertility stress. Furthermore, in the present sample, Cronbach’s *α* reached 0.84, confirming adequate internal consistency.

#### 3.4.3. Patient Health Questionnaire‐9 (PHQ‐9)

Assessment of depressive symptoms among female partners of infertile couples was conducted using the Patient Health Questionnaire‐9 (PHQ‐9). This instrument, validated globally including in Chinese populations, demonstrates excellent psychometric properties [32]. Participants rated each item on a 4‐point Likert scale (0 = not at all and 3 = nearly every day), resulting in total scores ranging from 0 to 27. Conventional scoring thresholds categorize symptoms as 0–5 (no depression), 6–9 (mild), 10–14 (moderate), 15–19 (moderately severe), and 20–27 (severe). Based on our sample distribution, moderate, moderately severe, and severe categories were merged into a single “moderate‐to‐severe depressive symptoms” group, while no depression and mild classifications remained separate. Instrument reliability was confirmed by a Cronbach’s *α* of 0.86, indicating strong internal consistency.

#### 3.4.4. Family Adaption, Partnership, Growth, Affection, and Resolve (Family APGAR)

The Family APGAR questionnaire was employed to evaluate participants’ satisfaction with family function. Developed by Smilkstein in 1978 [33], this instrument assesses family function across five dimensions: adaptability, partnership, growth, affection, and resolve. These dimensions reflect whether the participant feels that family members provide support, share problems, show affection, and work together in times of difficulty. In this study, the Family APGAR was used to reflect the participant’s overall perception of family function rather than being limited solely to the partner relationship. In the Chinese cultural context, this perception may be influenced by interactions within both the immediate family and the broader family network [34]. The questionnaire uses a 3‐point Likert scale: “often” (2 points), “sometimes” (1 point), and “hardly ever” (0 point). Total scores range from 0 to 10 points, indicating optimal family function; 4–6 points, suggesting moderate dysfunction; and 0–3 points, indicating severe dysfunction. In this study, the Cronbach’s *α* coefficient of the Family APGAR was 0.87, indicating high internal consistency.

#### 3.4.5. Infertility Stigma Scale (ISS)

The ISS, developed by Fu et al. [35], was used to assess infertility stigma among Chinese female partners of infertile couples. This scale has been widely applied in clinical settings. The ISS consists of 27 items across four dimensions: self‐deprecation (seven items), social withdrawal (five items), stigma from others (nine items), and stigma from family members (six items). Representative items reflect feelings of self‐blame, avoidance of social interaction, perceived negative judgments from others, and pressure or misunderstanding from family members. All items are positively scored using a 5‐point Likert scale (1 = strongly disagree and 5 = strongly agree). Total scores range from 27 to 135, with higher scores indicating greater infertility stigma. In this study, the Cronbach’s *α* coefficient of the ISS was 0.98, indicating excellent internal consistency.

### 3.5. Statistical Methods

Data were double‐entered and verified using Epidata 2.0 software to ensure accuracy. Statistical analyses were performed using R 4.4.1. For continuous variables conforming to a normal distribution, results were presented as mean ± standard deviation (M ± SD), and independent samples *t*‐tests or one‐way analysis of variance (ANOVA) were used for group comparisons. For non‐normally distributed continuous variables, the median and interquartile range [*M* (*p*25, *p*75)] were reported, and nonparametric tests were applied for group comparisons. Categorical variables were described using frequencies and percentages, and group comparisons were conducted using *χ*‐square tests. Spearman’s rank correlation analysis was used to evaluate the correlations between variables.

Structural equation modeling (SEM) and mediation effect testing were performed using AMOS 28.0. Model fit was evaluated based on the following criteria: a normalized *χ*‐square (*χ* ^2^/*df*) <3 indicated good fit, and 3–5 was acceptable; the comparative fit index (CFI) >0.95; the goodness‐of‐fit index (GFI) and the adjusted goodness‐of‐fit index (AGFI) ≥0.90; the normed fit index (NFI) >0.90; the root mean square error of approximation (RMSEA) <0.05; and standardized root mean square residual (SRMR) <0.08 [36–38]. These criteria were used to assess model adequacy and plausibility.

## 4. Results

### 4.1. Participant Characteristics

A total of 1294 female partners of infertile couples were enrolled. The mean age was 33.65 years (SD = 4.99). The majority were of Han ethnicity (*n* = 1092, 84.44%), and 1029 participants (75.66%) were registered residents of Hainan Province. Urban residence was reported by 745 women (57.57%). Educational level below bachelor’s level was observed in 874 participants (67.54%), and 1162 (89.80%) reported high self‐perceived economic pressure. Regarding infertility treatment duration, 520 women (54.65%) had undergone treatment for more than 6 months. With respect to mental health, 341 participants (26.35%) presented with depressive symptoms of varying severity. Subsequent analyses revealed statistically significant differences in depressive symptoms across native place, educational level, self‐perceived economic pressure, infertility treatment duration, and history of miscarriage (all *p* < 0.05). Details are provided in Table 1.

**Table 1:** Comparison of demographic characteristics across depressive symptom severity groups among female partners of infertile couples [*n* = 1294].

Independent variables	No depression [*n* = 953, 73.5%]		Mild depression [*n* = 231, 17.9%]		Moderate‐to‐severe depressive symptoms [*n* = 110, 8.5%]		*χ* ^2^	*p*
Age							0.192	0.825
Mean ± SD	33.67	5.037	33.49	4.756	33.83	5.129		
Ethnicity								
Han	808	84.78	198	85.70	86	78.20	5.500	0.233
Li	118	12.38	24	10.40	18	16.40		
Other ethnicities	27	2.840	9	3.90	6	5.50		
Residence							0.511	0.775
Urban	553	58.00	132	57.10	60	54.50		
Rural	400	42.00	99	42.90	50	45.50		
Native place							**4.606**	**0.032 ^∗^**
Local (Hainan)	772	81.00	175	75.80	82	74.50		
Nonlocal	181	19.00	56	24.20	28	25.50		
Educational level							**10.875**	**0.028 ^∗^**
Junior high school or below	340	35.70	62	26.80	48	43.60		
Senior high school or junior college	305	32.00	88	38.10	31	28.20		
Bachelor’s degree or above	308	32.30	81	35.10	31	28.20		
Self‐perceived economic pressure							**73.693**	**<0.001 ^∗∗∗^**
None	117	12.30	9	3.90	6	5.41		
Rarely	105	11.00	13	5.60	6	5.41		
Slight	436	45.80	92	39.80	36	32.43		
Moderate	183	19.20	80	34.60	33	29.73		
Severe	112	11.80	36	15.60	30	27.03		
Occupation							8.59	0.378
Unemployed	199	20.90	53	22.90	22	21.20		
Farmer	88	9.20	16	6.90	16	9.30		
Self‐employed	110	11.50	24	10.40	15	11.50		
Enterprise & public institution employer	266	27.90	66	28.60	33	28.20		
Others	290	30.40	72	31.20	24	29.80		
Spouse’s Occupation							13.73	0.089
Unemployed	74	7.80	19	8.20	11	10.00		
Farmer	104	10.90	19	8.20	19	17.30		
Self‐employed	141	14.80	30	13.00	21	19.10		
Enterprise and public institution employer	254	26.70	75	32.50	27	24.50		
Others	380	39.90	88	38.10	32	29.10		
Annual household income (RMB: yuan)							1.84	0.934
<30,000	199	20.90	45	19.50	26	23.60		
30,000 to 60,000	244	25.60	66	28.60	27	24.50		
60,000 to 90,000	185	19.40	40	17.30	20	18.20		
>90,000	325	34.10	80	34.60	37	33.60		
Infertility treatment duration							**7.13**	**0.028 ^∗^**
<6 months	433	45.44	83	35.93	45	40.91		
≥6 months	520	54.56	148	64.07	65	59.09		
History of miscarriage							**8.96**	**0.011 ^∗^ **
None	845	88.67	190	82.25	101	91.82		
Yes	108	11.33	41	17.75	9	8.18		
Causes of infertility							11.477	0.075
Female	397	41.70	397	35.50	397	50.00		
Male	97	10.20	97	7.80	97	4.50		
Both	158	16.60	158	18.20	158	16.40		
Unexplained	301	31.60	301	38.50	301	29.10		
Presence of children							3.27	0.195
No existing children	653	68.50	165	71.40	84	76.40		
≥1 children	300	31.50	66	28.60	26	23.60		

*Note:* Bold values indicate statistically significant differences (*p* < 0.05, chi‑square test).

^∗^ *p* < 0.05.

^∗∗^ *p* < 0.01.

^∗∗∗^ *p* < 0.001.

### 4.2. Spearman Correlation Analysis

Spearman correlation analysis revealed significant associations among fertility stress, infertility stigma, family function, and depressive symptoms (all *p* < 0.001). Specifically, fertility stress (*r* = 0.231, *p* < 0.001) and infertility stigma (*r* = 0.366, *p* < 0.001) were positively correlated with depressive symptoms, whereas family function (*r* = −0.254, *p* < 0.001) was negatively correlated with depressive symptoms. Additionally, fertility stress was positively correlated with infertility stigma (*r* = 0.584, *p* < 0.001) but negatively correlated with family function (*r* = −0.314, *p* < 0.001). Family function was also negatively correlated with infertility stigma (*r* = −0.301, *p* < 0.001). Details are presented in Table 2.

**Table 2:** Spearman correlation coefficient matrix among variables.

Variable	M	*p*25, *p*75	Depressive symptoms	Fertility stress	Family function
1 Depressive symptoms	3	1,6	1		
2 Fertility stress	143	126,159	0.231 ^∗∗^	1	
3 Family function	8	5,10	−0.254 ^∗∗^	−0.314 ^∗∗^	1
4 Infertility stigma	53	31,66	0.366 ^∗∗^	0.584 ^∗∗^	−0.301 ^∗∗^

*Note:* M, median; *p*25, *p*75, interquartile range.

^∗∗^ *p* < 0.01.

### 4.3. Direct Effect Model

**Figure 2:**
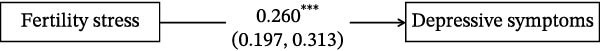
Path coefficients of the direct effect model. *Note:*  ^∗∗∗^ *p* < 0.01.

Building upon significant correlations among variables, this study employed a direct effect model to further examine the predictive effect of fertility stress on depressive symptoms in female partners of infertile couples. The model demonstrated adequate fit: *χ* ^2^/*df* = 2.342, *p* < 0.05; RMSEA = 0.032; SRMR = 0.025; GFI = 0.99; AGFI = 0.98; NFI = 0.97; CFI = 0.98 (see Figure 2). Path analysis revealed that fertility stress exerted a significant positive predictive effect on depressive symptoms (*β* = 0.260, *p* < 0.001). The direct effect size accounted for 12.7% of the variance (*R* ^2^ = 0.127), with the 95% confidence interval for the effect value (0.197, 0.313) excluding zero, confirming statistical significance of this pathway.

### 4.4. Parallel Mediation Model

**Figure 3:**
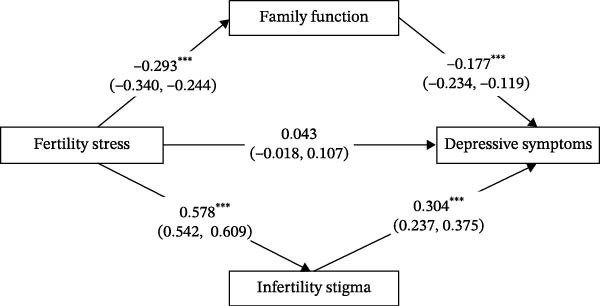
Path coefficients of the parallel mediation effect model. *Note:*  ^∗∗∗^ *p* < 0.01.

A parallel mediation model was further constructed to explore the influence of fertility stress on depressive symptoms in female partners of infertile couples through infertility stigma and family function. In this model, depressive symptoms served as the dependent variable, fertility stress as the independent variable, and infertility stigma and family function as parallel mediators. Statistically significant demographic covariates (e.g., place of origin, education level, and economic pressure) were controlled for in the model. The model demonstrated good fit with the following indices: *χ* ^2^/*df* = 3.617 (*p* < 0.001), RMSEA = 0.045, SRMR = 0.033, GFI = 0.99, AGFI = 0.97, NFI = 0.96, CFI = 0.97 (see Figure 3).

Analysis of the constructed model revealed that the direct effect of fertility stress on depressive symptoms among female partners of infertile couples was nonsignificant (*β* = 0.043, *p* > 0.05). However, fertility stress exerted significant indirect effects on depressive symptoms through the mediators of infertility stigma and family function. Specifically, the mediating effect via family function was significant (*β* = 0.052, *p* < 0.001), as was the mediating effect via infertility stigma (*β* = 0.176, *p* < 0.001). These indirect effects accounted for 22.81% and 77.19% of the total indirect effect, respectively. The total indirect effect was statistically significant (*β* = 0.228, *p* < 0.001). The path coefficients of the parallel mediation analysis are presented in Table 3.

**Table 3:** Path coefficients of parallel mediation analysis.

Path	Standardized coefficient	*Z*	SE	95%CI	
Total effect	0.271 ^∗∗∗^	9.34	0.029	0.213	0.325
Direct effect	0.043	1.34	0.032	−0.018	0.107
Indirect effects					
(1) Fertility stress → family function → depressive symptoms	0.052 ^∗∗∗^	5.20	0.010	0.033	0.072
(2) Fertility stress → infertility stigma → depressive symptoms	0.176 ^∗∗∗^	8.00	0.022	0.135	0.220
Total indirect effect: (1) + (2)	0.228 ^∗∗∗^	10.36	0.022	0.185	0.270
Comparison of parallel mediation effects: (1) − (2)	−0.124 ^∗∗∗^	−4.96	0.025	−0.176	−0.077

*Note: Z*, standard score; SE, standard error; 95% CI, 95% confidence interval.

^∗∗∗^ *p* < 0.001.

Further analysis revealed distinct mediating pathways through which family function and infertility stigma operated in the relationship between fertility stress and depressive symptoms. Specifically, increased fertility stress was associated with diminished family function, which, in turn, may exacerbate the risk of depressive symptoms among female partners of infertile couples. Concurrently, higher fertility stress was associated with greater infertility stigma, which in turn was associated with more severe depressive symptoms. A comparison of the standardized coefficients for the two mediating pathways yielded a significant difference (difference = −0.124, *p* < 0.001; 95% CI: −0.285 to −0.135). The 95% confidence interval did not include zero, indicating a significant difference between the two mediating effects. This difference demonstrates that the mediating effect of infertility stigma in the association between fertility stress and depressive symptoms was significantly stronger than that of family function.

## 5. Discussion

### 5.1. Incidence and Influencing Factors of Depression Symptoms

#### 5.1.1. Incidence of Depressive Symptoms

This study investigated 1294 female partners of infertile couples in Hainan Province, China. The results revealed that 26.35% of participants exhibited varying degrees of depressive symptoms. This prevalence rate is comparable to domestic studies utilizing the same assessment instrument, such as the 25.4% depression rate reported by Chen and Bo [39]. However, it is higher than the 23.03% depression prevalence derived from a national database in China by Hong et al. [40].

Internationally, a systematic review and meta‐analysis conducted by Kiani et al. [41] indicated that the prevalence of depressive symptoms among female partners of infertile couples is generally elevated worldwide, with reported rates ranging from 12.2% to 25.6%. This variation is attributed to differences in study regions, screening tools, and sample characteristics. The review highlighted significant disparities in prevalence rates across countries and regions, exemplified by rates of 15.7% in Switzerland, 25.6% in Nigeria, and 12.2% in Vietnam. These differences underscore the critical role of sociocultural contexts, economic support systems, and public health resources in influencing the mental health of these women. Therefore, it is particularly important to conduct localized studies in the context of Chinese social culture. Such studies can not only reveal the mental health characteristics of female partners of infertile couples in China but also provide useful contextual evidence for future psychosocial support and intervention development.

#### 5.1.2. Influencing Factors of Depression Symptoms

This study found that depressive symptoms in female partners of infertile couples are influenced by various sociodemographic factors. Regarding place of origin, those with nonlocal household registration in Hainan had a higher risk of moderate‐to‐severe depressive symptoms, which may be associated with regional cultural differences and a lack of sense of belonging [42]. Notably, in contrast to previous findings, female partners with a bachelor’s degree or above were more likely to experience mild depressive symptoms. This result is consistent with a Chinese study by Li et al. [43], which reported that higher education level was associated with greater emotional disorders among perimenopausal women. A possible explanation is that highly educated women often face the dual expectation of being a “career elite and perfect mother,” and infertility may disrupt their sense of self‐continuity; moreover, they tend to value long‐term planning, and delayed childbearing may lead to “opportunity cost anxiety” [43]. Furthermore, higher self‐rated economic pressure was associated with greater severity of depressive symptoms, which may be attributed to the high cost of treatment (e.g., the annual cost of polycystic ovary syndrome exceeding $15 billion [44]) that exacerbates financial burden and psychological stress [45, 46]. In addition, longer duration of assisted reproductive treatment was associated with a higher risk of depressive symptoms, as prolonged treatment may impose a cumulative burden of repeated hope‐disappointment cycles, financial strain, and chronic uncertainty, gradually eroding emotional resilience [47, 48]. Finally, a history of miscarriage in female partners of infertile couples was associated with a higher risk of depressive symptoms, possibly because miscarriage impairs self‐worth, causes physical burden, and intensifies concerns about future pregnancies [49–52].

### 5.2. Interpretation of Multiple Mediating Effects on Depressive Symptoms

This study further examined the pathways linking fertility stress and depressive symptoms using SEM. The results supported a full mediation pattern, in which the association between fertility stress and depressive symptoms operated entirely through infertility stigma and family function, with infertility stigma emerging as the primary mediator. This finding has important theoretical relevance and may offer useful implications for understanding the psychosocial pathways linking fertility stress and depressive symptoms.

Infertility stigma plays a critical role in the mediating pathway, which may originate from individuals’ internalization of social expectations toward childbearing. When female partners of infertile couples fail to achieve the goal of childbearing, they often experience a “sense of failure” or a deviation from social roles, thereby developing intense feelings of shame. Such emotions can further affect individuals’ self‐worth evaluation, social behaviors, and emotional states, ultimately increasing the risk of depressive symptoms [53]. In the context of traditional Chinese culture, childbearing is endowed with significant family and social meanings, and women’s reproductive role is particularly prominent [54]. For instance, the Li and Han cultures in Hainan emphasize matrilineal inheritance and bloodline continuity, where women’s fertility is regarded as an important symbol of family responsibility and social recognition [55, 56]; this can further strengthen the formation of infertility stigma. Psychological stress in this cultural context not only increases emotional distress but also becomes a key factor influencing behavioral patterns (e.g., social avoidance) and social adaptability. It should also be noted that the relationship between fertility stress and infertility stigma may not be strictly unidirectional. Although the present model was specified on theoretical grounds, infertility stigma may also intensify stress perceptions. Because this study was cross‐sectional, alternative directional or reciprocal relationships could not be tested and should be examined in future longitudinal research.

Meanwhile, family function, as another important mediating pathway, plays a key role in buffering fertility stress and supporting psychological adaptation. A sound family function can provide emotional support, problem‐solving resources, and self‐efficacy for female partners of infertile couples, thereby reducing their level of depressive symptoms [57, 58]. However, when fertility stress affects spousal communication, family roles, or emotional bonds, impaired family function may lead individuals to feel isolated, helpless, and resource‐deficient. These family factors not only limit psychological adjustment abilities but also further weaken individuals’ coping strategies when facing infertility‐related difficulties, thereby increasing the risk of depression [59, 60]. Therefore, fertility stress may be indirectly associated with depressive symptoms through poorer family function, making family function an important target in the design of psychological interventions.

Integrating the findings above, the present mediation model revealed that fertility stress experienced by female partners of infertile couples was not directly associated with depressive symptoms after accounting for the mediating effects of infertility stigma and family function. Instead, its association with depressive symptoms operated entirely through these two parallel mediators. This finding does not contradict the extensive literature demonstrating a direct positive relationship between general stress and depression; rather, it provides a more nuanced understanding of how fertility stress specifically affects depressive symptoms in this population. Previous studies have typically examined direct associations, whereas our study identified infertility stigma and family function as key intervening variables. Together, these findings support the hypothesized parallel mediation model. This outcome not only addresses a theoretical gap in research related to fertility stress but also suggests that future psychosocial support efforts may benefit from considering both infertility stigma and family function. Specifically, future intervention development may benefit from attending to both the psychological dimension of infertility stigma and the social support dimension reflected in family function.

### 5.3. Suggestions for Public Health Intervention

Based on the finding that infertility stigma and family function are two parallel mediators linking fertility stress to depressive symptoms, we propose intervention recommendations targeting these two pathways separately.

**Reducing infertility stigma.** In the Chinese cultural context, women with infertility often experience intense shame and self‐doubt due to unmet expectations of the reproductive role. Interventions should first aim to reduce stigma. At the medical system level, educational support should be provided to infertile women and their partners, covering infertility etiology, treatment pathways, and emotional coping, to correct misconceptions and reduce self‐stigma [61–63]. Such education should be extended to family members as changes in family members’ understanding may help alleviate the interpersonal stigma experienced by the patient. At the societal level, public education and awareness initiatives can improve social understanding and reduce prejudice [64]. A localized example is Liu et al. [65], who conducted a low‐cost online expressive art intervention for 80 western Chinese infertile women, which alleviated depressive symptoms within 4 weeks by easing stigma‐related isolation.

**Improving family function.** Fertility stress indirectly increases the risk of depression by impairing family function. Therefore, interventions should shift from traditional individual‐only support to family‐integrated support, designing programs to enhance couple communication, family intimacy, and adaptability. For example, a randomized controlled trial by Zhao et al. [66] implemented cognitive behavioral group psychological nursing combined with family members’ participation among infertility patients undergoing assisted reproductive technology. The intervention significantly improved family function and reduced stress, anxiety, and depression [66].

**Interventions targeting both stigma and family function.** Furthermore, some interventions have been shown to simultaneously address both pathways. For example, Lin et al. [64] developed a localized family‐oriented intervention for Chinese women undergoing infertility treatment, combining teach‐back health education and short‐video guidance delivered via a local social platform and invited family members to participate in joint learning sessions. This intervention not only improved family intimacy and adaptability but also reduced infertility stigma, thereby alleviating depressive symptoms [64].

In summary, psychological interventions should simultaneously address both stigma reduction and family function improvement and can be implemented synergistically at three levels: medical system (educational support and couple counseling), societal (public campaigns to reduce discrimination), and family (skills training to enhance communication and support). Such a multilevel, dual‐target intervention strategy may more effectively reduce the risk of depression in this population.

## 6. Limitations and Strengths

This study has several limitations. First, the cross‐sectional design precludes conclusions about temporal ordering or causality, and future longitudinal studies are needed to further examine the mediating pathways identified in this study [67]. Second, only female partners of infertile couples were included. Although this focus is meaningful given the substantial infertility‐related stigma and psychological burden often experienced by women in the Chinese cultural context, infertility is typically experienced at the couple level. Therefore, family function in the present study was assessed only from the female partner’s perspective and may not fully capture dyadic interactions or the influence of male partners on stress, stigma, and psychological adjustment. Third, participants were recruited from two Grade A tertiary public hospitals in Hainan Province, China, which may limit the generalizability of the findings to other settings or regions. In addition, anxiety symptoms were not assessed, and detailed ART and infertility‐related clinical characteristics were not fully captured, including treatment status at the time of questionnaire completion and specific assisted reproductive treatment modalities. These factors may have limited a more comprehensive understanding of psychological distress in this population. Future studies should include participants from other regions and multicenter settings, incorporate couple‐level information, and simultaneously consider treatment‐related characteristics as well as both anxiety and depressive symptoms.

Notwithstanding these limitations, this study also has several strengths. First, participants were recruited from two major reproductive medicine centers in Hainan Province, providing a relatively large clinical sample of female partners of infertile couples seeking treatment. This helps enhance the clinical relevance of the findings. Second, this study examined infertility stigma and family function simultaneously within a parallel mediation framework in the Chinese cultural context, thereby extending current understanding of the psychosocial pathways linking fertility stress and depressive symptoms. These findings may offer useful implications for future psychosocial support and intervention development for infertile women.

## 7. Conclusions

This study revealed a high prevalence of depressive symptoms among female partners of infertile couples in China. The findings support a parallel mediation model in which the association between fertility stress and depressive symptoms operated through infertility stigma and family function. This finding elucidates the complexity and cultural specificity of the underlying psychological mechanism. Building on these findings, future psychosocial support efforts may benefit from paying attention to infertility stigma, family function, and broader social support systems. These areas may be considered in future intervention development and service planning for this population. Collectively, the findings provide insight into the psychosocial processes associated with depressive symptoms among female partners of infertile couples and may offer a useful basis for future research and culturally sensitive support planning.

## Author Contributions

Yingqi Li performed statistical analysis and drafted the manuscript. Ying Wang, Meihong Wang, and Hua Yang contributed to data collection and revision of the manuscript. Lei Qiu conceived, designed and supervised the study as corresponding authors. All other content, including study design, data collection, analysis, interpretation, and manuscript drafting, was performed solely by the authors.

## Funding

This project was funded by the Open Competition (OC) Program of the China Medical Board (CMB) (Grant CMB‐OC 22‐474) and the 2024 Hainan Provincial Graduate Student Innovative Research Project organized by The Education Department of Hainan Province (Grant Qhys2024‐439).

## Disclosure

All authors have read and approved the final manuscript. The authors are fully responsible for the final content of the manuscript. The funder had no role in study design, data collection and analysis, decision to publish, or preparation of the manuscript.

## Ethics Statement

In accordance with the Declaration of Helsinki, the study has received approval from the Ethics Committee of Hainan Women and Children’s Medical Center and the Infertility Center of the First Affiliated Hospital of Hainan Medical University (2023‐KYL‐075). Our study participants included women from infertile couples from Hainan Province who came to the reproductive departments of the two hospitals for treatment. This form provided comprehensive information about the study’s procedures, its objectives, and the rights of the participants, including their right to withdraw from the study at any time without any adverse consequences. Participants were informed in advance that completing the questionnaire would be considered their consent to participate in the study, with the need for a physical signature. Given that our study involved only adult participants, obtaining consent from parents or guardians was not applicable.

## Conflicts of Interest

The authors declare no conflicts of interest.

## Data Availability

The data that support the findings of this study are available from the corresponding author upon reasonable request.
